# A Genealogical Interpretation of Principal Components Analysis

**DOI:** 10.1371/journal.pgen.1000686

**Published:** 2009-10-16

**Authors:** Gil McVean

**Affiliations:** Department of Statistics, University of Oxford, Oxford, United Kingdom; University of Chicago, United States of America

## Abstract

Principal components analysis, PCA, is a statistical method commonly used in population genetics to identify structure in the distribution of genetic variation across geographical location and ethnic background. However, while the method is often used to inform about historical demographic processes, little is known about the relationship between fundamental demographic parameters and the projection of samples onto the primary axes. Here I show that for SNP data the projection of samples onto the principal components can be obtained directly from considering the average coalescent times between pairs of haploid genomes. The result provides a framework for interpreting PCA projections in terms of underlying processes, including migration, geographical isolation, and admixture. I also demonstrate a link between PCA and Wright's *f_st_* and show that SNP ascertainment has a largely simple and predictable effect on the projection of samples. Using examples from human genetics, I discuss the application of these results to empirical data and the implications for inference.

## Author Summary

Genetic variation in natural populations typically demonstrates structure arising from diverse processes including geographical isolation, founder events, migration, and admixture. One technique commonly used to uncover such structure is principal components analysis, which identifies the primary axes of variation in data and projects the samples onto these axes in a graphically appealing and intuitive manner. However, as the method is non-parametric, it can be hard to relate PCA to underlying process. Here, I show that the underlying genealogical history of the samples can be related directly to the PC projection. The result is useful because it is straightforward to predict the effects of different demographic processes on the sample genealogy. However, the result also reveals the limitations of PCA, in that multiple processes can give the same projections, it is strongly influenced by uneven sampling, and it discards important information in the spatial structure of genetic variation along chromosomes.

## Introduction

The distribution of genetic variation across geographical location and ethnic background provides a rich source of information about the historical demographic events and processes experienced by a species. However, while colonization, isolation, migration and admixture all lead to a structuring of genetic variation, in which groups of individuals show greater or lesser relatedness to other groups, making inferences about the nature and timing of such processes is notoriously difficult. There are three key problems. First, there are many different processes that one might want to consider as explanations for patterns of structure in empirical data and efficient inference, even under simple models can be difficult. Second, different processes can lead to similar patterns of structure. For example, equilibrium models of restricted migration can give similar patterns of differentiation to non-equilibrium models of population splitting events (at least in terms of some data summaries such as Wright's 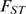). Third, any species is likely to have experienced many different demographic events and processes in its history and their superposition leads to complex patterns of genetic variability. Consequently, while there is a long history of estimating parameters of demographic models from patterns of genetic variation, such models are often highly simplistic and restricted to a subset of possible explanations.

An alternative approach to directly fitting models is to use dimension-reduction and data summary techniques to identify key components of the structure within the data in a model-free manner. Perhaps the most widely used technique, and the most important from a historical perspective, is principal components analysis (PCA). Technical descriptions of PCA can be found elsewhere, however, its key feature is that it can be used to project samples onto a series of orthogonal axes, each of which is made up of a linear combination of allelic or genotypic values across SNPs or other types of variant. These axes are chosen such that the projection of samples along the first axis (or first principal component) explains the greatest possible variance in the data among all possible axes. Likewise, projection of samples onto the second axis maximizes the variance for all possibles axes perpendicular to the first and so on for the subsequent components. Typically, the positions of samples along the first two or three axes are presented, although methods for obtaining the statistical significance of any given axis have been developed [1]. Beyond being non-parametric, PCA has many attractive properties including computational speed, the ability to identify structure caused by diverse processes and its ability to group or separate samples in a striking visual manner; for example, see [2]. PCA has also become widespread in the analysis of disease-association studies where the inclusion of the locations of samples on a limited number of axes as covariates can be used in an attempt to control for population stratification [3].

Although PCA is explicitly a non-parametric data summary, it is nevertheless attractive to attempt to use the projections to make inferences about underlying events and processes. For example, dispersion of sample projections along a line is thought to be diagnostic of the samples being admixed between the two populations at the ends of the line, though these need not always be present [1], while correlations between principal components and geographical axes have been interpreted as evidence for waves of migration [4],[5]. However, while simulation studies have shown that such patterns do occur when the inferred process has acted [1],[6], they can also be caused by other processes or even statistical artefacts. For example, clines in principal components result not just from waves of expansion, but also recurrent bottlenecks, admixture and equilibrium models of spatial structure [6]–[11].

In this paper I develop a framework for understanding how PCA relates to underlying processes and events. I show that the expected location of samples on the principal components can, for single nucleotide polymorphism (SNP) data, be predicted directly from the pairwise coalescence times between samples. Because it is often relatively easy to obtain analytical or numerical solutions to expected coalescence times under explicit population genetics models, it is also possible to obtain expressions for the PCA projections of samples under diverse scenarios, including island models, models with isolation and founder events and historical admixture. The result also highlights some key limitations of PCA. For example, it follows that PCA cannot be used to distinguish between models that lead to the same mean coalescence times (for example models with migration or isolation). Furthermore, PCA projections are strongly influenced by uneven sampling. Using examples from human genetics I discuss the implications of these results for making inferences from PCA of genetic variation data.

## Results

### PCA describes structure in the matrix of pairwise coalescence times

In this section I provide a brief summary of how PCA is carried out and describe the key result concerning the relationship between PCA and average coalescence time. In what follows I assume that 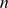 haploid individuals have been sequenced with complete accuracy (diploid samples and the influence of SNP ascertainment will be discussed later). The only polymorphisms present are biallelic SNPs that are the result of a single historical mutation. Let 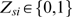 be the allelic state for individual *i* at locus *s* (here I assume that the ancestral allele is defined as 0 and the derived allele as 1, however the following also applies for any coding, for example where the minor allele is coded as 1). After removing monomorphic sites the data, 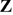, consist of an 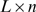 binary matrix (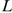 is the number of SNPs). In PCA, the first step is to zero-centre the data, so as to create a new matrix, X, where
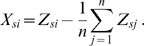
(1)At this stage, the data rows are often normalized so as to have equal variance, however, it is assumed that this is not the case (in practice normalization has little effect for SNP data, though will tend to up-weight the influence of rare variants). Each individual sample can be thought of as representing a point in *L*-dimensional space, where each dimension (or axis) represents a single SNP. The goal of PCA is to find a new set of orthogonal axes (the principal components), each of which is made up from a linear combination of the original axes, such that the projection of the original data onto these new axes leads to an efficient summary of the structure of the data. More formally, PCA defines a stretch and rotation transformation, expressed through the matrix 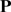, such that application of 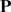 to the original data (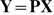) leads to transformed data with the following properties.

1. The transformed data matrix, 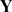, has the same dimensions (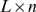) as the original data and the mean of each row is zero.
2. The value associated with a given individual in 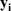, the *i*th *row* of 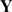, represents the individual's position or projection on the *i*th principal component.
3. The correlation between any two rows of 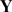 is zero.
4. The sum of the variances of the rows equals the variance in the original data.
5. The variances of the rows are monotonically decreasing.
6. The variance of the first row is the largest of any possible projection of the original data on a linear combination of the SNPs.

The principal components can be obtained directly by finding the eigenvectors of the covariance matrix
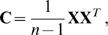
(2)such that the *i*th principal component (the *i*th *row* of 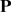, 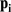) is the *i*th eigenvector of 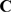. However, because 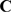 (of dimension 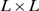) can be very large for genome-wide SNP data sets, it can be more convenient to use singular value decomposition (SVD) to find the principal components and individual projections. SVD, which exists for any 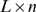 real matrix (where 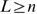) rewrites the original data in terms of three other matrices
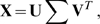
(3)where 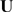 is an orthogonal matrix (i.e. the dot-product between any two columns is zero) of dimension 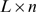, 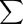 is a diagonal matrix of dimension 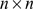 and 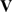 is another orthogonal matrix of dimension 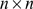. This is achieved by setting 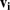, the *i*th *column* of 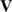, to be the *i*th eigenvector of the matrix
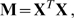
(4)

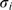
, the *i*th diagonal entry of 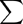 to be the square root of the corresponding eigenvalue and 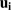, the *i*th *column* of 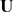, to be the vector
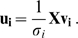
(5)PCA and SVD are, through construction, intimately related. Specifically, the projection of samples along the *i*th principal component is given by 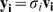 (note this is the *i*th *row* of 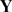 and the *i*th *column* of 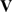) and the *i*th principal component is 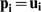. For typical population genetics data sets, eigenvalue analysis of the matrix 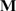 (of dimension 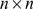) is computationally simpler than analysis of the matrix 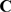 (typically hundreds or thousands of samples have been genotyped at hundreds of thousands or millions of SNPs). The above construction results in the projection of samples on the PCs being influenced by the number of SNPs (e.g. repeating the analysis on a data set in which every SNP is included twice will lead to projections that are a factor 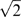 larger than previously). To correct for this, consider a slightly different definition of the matrix 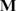:
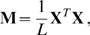
(6)which is equivalent to dividing the data matrix by the square-root of the number of SNPs. It is worth noting that 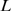 may either be a random variable as in the case of sequencing, or a fixed variable, as in the case of genotyping. Here, it will be treated as a fixed variable, though in practice this is of little importance.


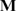
 is a stochastic matrix. However, it is possible to learn about the key structural features of 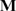 by considering its expectation. From above, it follows that
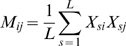
(7a)

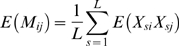
(7b)

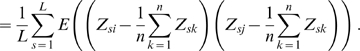
(7c)Assuming that sites are identical in distribution (though not necessarily independent) the subscript 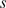 can be dropped to give
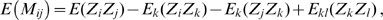
(8)where the terms such as 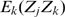 indicate the expectation (for sample 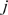) is averaged over all individuals 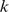 in the sample (note this includes self); i.e. 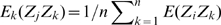. Because 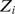 is either 0 or 1, the four terms in Equation 8 can be thought of as:

1. The probability that samples 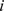 and 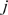 both carry a derived mutation at a randomly chosen locus conditional on the locus being polymorphic in the sample.
2. The probability that sample 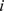 and another randomly chosen sample 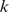 (which may include either 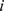 or 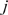) both carry the derived mutation at a randomly chosen locus.
3. The probability that sample 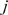 and another randomly chosen sample 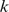 (which may include either 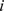 or 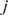) both carry the derived mutation at a randomly chosen locus.
4. The probability that two samples, 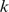 and 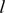, chosen at random with replacement both carry the derived mutation at a randomly chosen locus.

**Figure 1:**
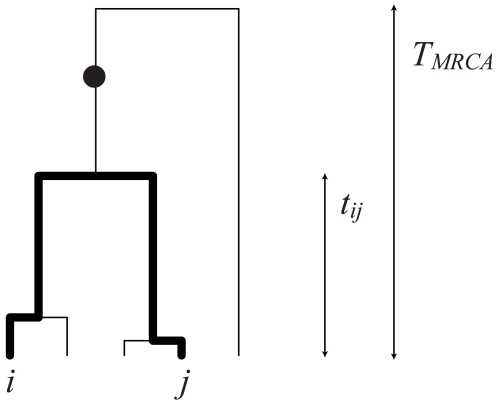
Genealogical statistics. The chart shows a genealogical tree describing the history of a sample of size five. Two samples, 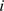 and 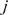, will share a derived mutation (indicated by the circle) if it occurs on the branch between their most recent common ancestor and the common ancestor of the whole sample. The length of this branch is 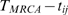.

In the case of a low mutation rate, where polymorphic sites are the result of a single historical mutation, expressions can be obtained for the above quantities in terms of features of the genealogical tree [12]–[14]. Figure 1 shows how the probability of two samples both carrying a mutation depends on their time to a common ancestor relative to the time to the common ancestor of the whole sample. Let 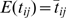 be the expected coalescence time for samples 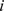 and 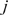, 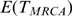 be the expected time to the most recent common ancestor of the sample, and 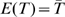 be the expected total branch length in the tree. The probability that two samples share a derived mutation (conditional on the site being segregating) is given by
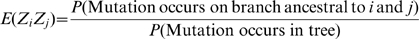
(9a)

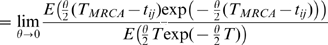
(9b)

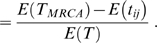
(9c)By writing similar expressions for the other terms in Equation 8 it follows that
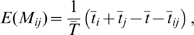
(10)where 
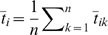
 and 
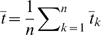
. Note that these expressions include coalescence with self where the coalescence time is always zero; i.e. 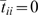. In short, the expectation of the matrix whose eigenvectors give the projections of samples on the principal components can be written in terms of the mean coalescence times for pairs of samples. It is worth noting that 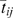 (and the related quantities) can be interpreted either as the expected coalescence time under some model or else the average realized coalescent time across the genome. The difference between these quantities can be important in some settings, such as admixture models (see below).

For diploid individuals the genotypic value for an individual at a given SNP is typically given by the sum of the allelic values; i.e. 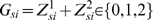, where the superscripts indicate the two alleles. By following the same argument as above it can be shown that for genotype data
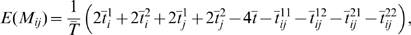
(11)where the superscripts again indicate the relevant allele in each individual. In the following I will assume that data consist of haplotypes, however Equation 11 makes it clear that essentially identical results will hold for genotype data.

### An example using two geographically separated populations

**Figure 2:**
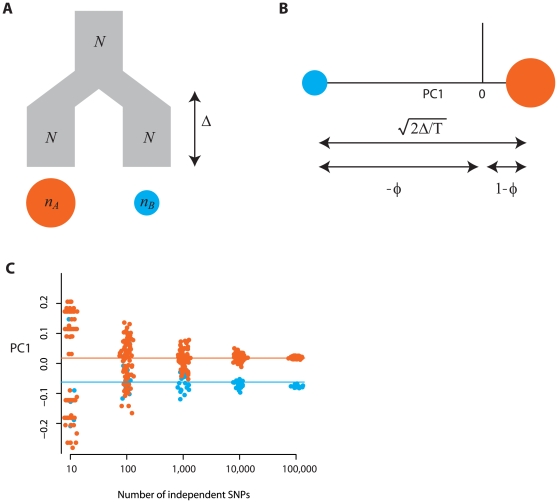
Principal component analysis of two populations. (A) Consider a sample of 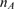 individuals from population A (indicated by the red circle) and 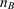 from population B (indicated by the blue circle), where the two populations have the same effective population size of 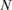 and are both derived from a single ancestral population, also of size 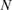, with the split happening a time 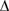 in the past. (B) The expected locations of these two sets of samples on the first PC is defined by the time since divergence (the Euclidean distance between the samples is 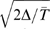) (see text for definitions) and the relative sample size from the populations, with the larger sample lying closer to the origin. Defining 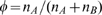, the relative location of the two populations on the first PC are 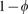 for samples from population A and 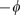 for samples from population B (note that the sign is arbitrary). (C) To investigate the effect of finite genome size simulations were carried out for the model shown in part A with 80 genomes sampled from population A, 20 from population B and a split time of 0.02 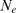 generations (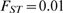) and between 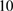 and 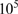 SNPs. Lines indicate the analytical expectation. A jitter has been added to the x-axis for clarity. Note that the separation of samples with 10 SNPs does not correlate with population and simply reflects random clustering arising from the small numbers of SNPs.

The implication of Equation 10 is that if the structure of pairwise coalescence times in a given data set can be understood, then the projection of the samples on the principal components can be predicted directly. Two illustrate this idea consider the simple model of a population split shown in Figure 2A. Under this model the expected coalescence time for pairs of samples within either population is 1 (in units of 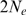 generations) and the expected coalescence time for pairs of samples from different populations is 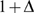, where 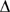 is the age of the population split (also in units of 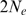 generations). Suppose of the total sample size 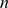, a fraction 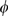 are from population A. Define 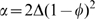 and 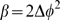, it follows that for large 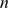, 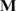 has a simple block structure;
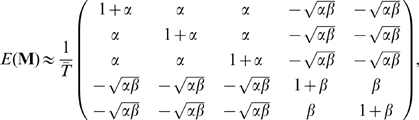
(12)where the first 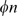 rows and columns represent the samples from population A (here, for example, three samples from A and two from B are shown). What will the leading eigenvalue and associated eigenvector be for a matrix with this kind of block structure? Although it is simple to obtain eigenvectors numerically, it is also worth having some intuition about what they represent. Through the construction of SVD it follows that the leading eigenvector, 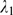 and eigenvector, 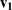, are those that, through Equation 3, provide the *best* approximation to the original data in terms of least-squares error. Equivalently, the matrix 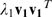 is the best least-squares approximation to 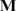. Intuitively, the original data is well approximated by the average allele frequency in each population and the the block structure of 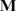 can be recovered by clustering samples from the two populations either side of the origin in 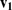. More formally, it can be shown that
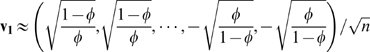
(13a)

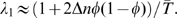
(13b)Assuming that 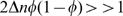 , the projection of the samples on the first principal component is given by the vector
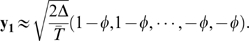
(14)Note that the sign of the projections is arbitrary. This result implies that the Euclidean distance between samples from the two populations on the first principal component will be 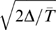 and their position relative to the origin is determined by the relative sample size, with the larger sample lying closer to the origin. Figure 2B shows the expected projection of samples.

These results refer explicitly to the expected value of 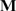. However, it is also important to know whether stochasticity resulting from the finite size of the genome has a significant effect on the results. Theoretical work on the nature and size of the first principal component in random matrices [15],[16] has identified a critical signal to noise ratio below which the true structure of the signal cannot be recovered. In the context of a two-population model this equates to 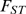 being greater than 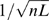 [1]. For example, with a sample size of 100 and 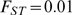, the threshold is 100 SNPs. Simulations were carried out for different numbers of independent SNPs (Figure 2C). As expected, for 10 or 100 SNPs PCA fails to separate samples from the two populations, while for 1,000 SNPs or more samples from the two populations are distinct on the first PC and centre around the theoretical expectation.

### PCA cannot distinguish between alternative models that have the same effect on mean coalescence time

A direct consequence of Equation 10 is that PCA predominantly reflects structure in the expected (or mean realized coalescent) time. Consequently, any two demographic models that give the same structure of expected coalescence times will also give the same projections. To illustrate this result, consider a fully general model with two homogeneous populations where the expected coalescence time for two samples from population A is 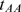, the expected coalescence time for two samples from population B is 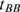 and the expected coalescence time for one sample from each population is 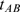. Define 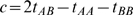, 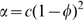 and 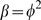. It can be shown that
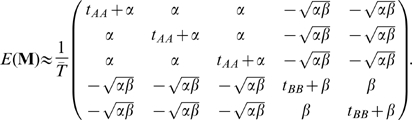
(15)Again, only three samples from population A and two from population B are shown. For large 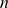, the leading eigenvalue and corresponding eigenvector of the above matrix are respectively
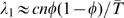
(16a)

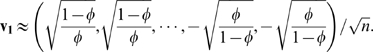
(16b)Consequently, the projection of the samples on the first principal component is given by the vector
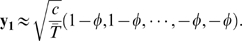
(17)Comparison of Equations 14 and 17 shows that the Euclidean distance between samples from the two populations on the first PC is a function of the difference between cross-population and within-population coalescence times and that the positioning of the populations relative to the origin simply reflects their relative sample size (as for the simpler two-population model). Consequently, any two models that give the same value of 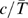 will give the same expected projections of samples on the first PC.

One connection that is worth exploring further is the link between the results shown here and those of Slatkin [12] concerning 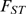. Slatkin showed that
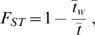
(18)where 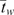 is the average coalescence time for pairs of samples from the same population and 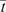 is the average coalescence time across all pairs of samples. In the notation used above it can be shown that
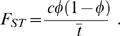
(19)Now consider the PCA projection. The variance along the first axis is 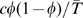. The total variance in the sample is 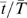. Consequently, the fraction of the total variance explained by the first PC is equal to 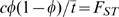. Given that 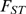 is defined as the fraction of the total variance that is explained by between-population differences this result is not surprising. Nevertheless, the result demonstrates a simple relationship between the Euclidean distance of populations in PCA space and 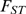, at least in the case of two populations.

### Uneven sampling has a strong influence on PCA projections

**Figure 3:**
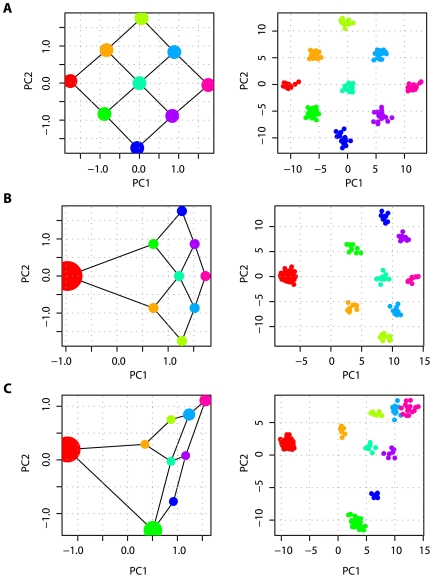
The effect of uneven sampling on PCA projection. PCA projection of samples taken from a set of nine populations arranged in a lattice, each of which exchanges migrants at rate 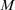 per 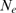 generations with each adjoining neighbour, leads to a recovery of the migration-space if samples are of equal size (A), or a distortion of migration-space if populations are not equally represented (B,C). In each part the left-hand panel shows the analytical solution (the area of each point represents the relative sample size) with migration routes illustrated while the right-hand panel shows the result of a simulation with a total sample size of 180 and 10,000 independent SNP loci. All examples are for 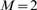.

As has been shown previously [11], PCA projections can be strongly influenced by uneven sampling from a series of populations. The results described here provide an explanation. First, from Equation 10 it can be seen that the the matrix 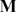 is influenced by the relative sample size from each population through the components 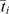. For instance, even if all populations are equally divergent from each other, those for which there are fewer samples will have larger values of 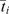 because relatively more pairwise comparisons are between populations. Second, even if the entries of 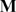 were not influenced by the relative sample size, its eigenvectors will be, simply because relative sample size will influence the structure of the genetic variance in the sample (see Figure 2). The influence of uneven sample size can be to bias the projection of samples on the first few PCs in unexpected ways, for example, where there is spatial structure to genetic variation. Consider a lattice arrangement of populations with equal migration between neighbouring populations. For this arrangement it is possible to obtain analytical expressions for the expected coalescence time for pairs of samples from the different populations (results not shown) and hence the matrix 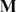 (up to an unknown scaling factor) and subsequently the projection of samples on the first few PCs under different assumptions about sample size and migration rate. If sample sizes from the different populations are equal, the spatial arrangement of the populations on the first two PCs mimics the structure of the migration matrix (Figure 3A). However, sample sizes differ between populations the effect is to distort the projection space (Figure 3B and 3C). This distortion of PC-space relative to the structure of the migration matrix is problematic for interpreting the location of samples on PCs. Sub-sampling from populations to achieve more equal representation, as in [2], is the only way to avoid this problem.

### The projection of admixed individuals onto existing axes directly identifies admixture proportions

The principal components identified through PCA can be used to project not just those samples from which the PCs were obtained, but also additional samples. The appeal of such analyses is that it enables the analysis of structural features identified in one data set to be transferred to another. For example, where data from two source populations and a set of possibly admixed samples are available, projection of the admixed samples onto the axes defined by the source populations can identify the extent of mixed ancestry. The advantage of this approach rather than simply performing PCA on all samples together is that other structural features within the admixed samples (e.g. admixture from a third population or relatedness) will have little influence on the projection. In the light of the above results showing how the PCA projection of samples can be interpreted in terms of coalescence times, it is interesting to ask how the the projection of additional samples onto the same axes also relates to coalescence times.

Consider the case of the general two-population model where the positions of the samples on the first PC are 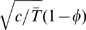 for samples from population A and 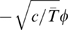 for samples from population B. The first PC can be obtained as in Equation 5. For a given SNP, 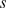, the expected loading for the first PC, 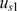, is therefore
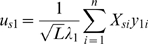
(20a)

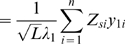
(20b)

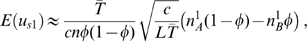
(20c)where 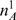 is the number of samples carrying the derived allele in population A. By writing 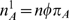 and 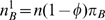, such that 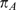 and 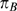 are the frequencies of the derived alleles in populations A and B respectively, it follows that
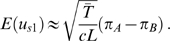
(21)The expected location of an additional sample, 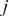, on the first PC is therefore
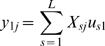
(22a)

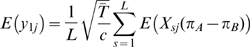
(22b)

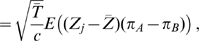
(22c)where 
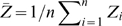
 (note this does not include the additional sample 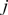). Again, the subscript 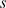 has been dropped by assuming that sites are identical in distribution. By noting that 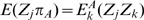, where the expectation is over those samples from population A, it follows that similar arguments to those above can be made to relate the quantities in Equation 22 to coalescent times. Define 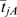 as the average coalescent time between the additional sample and all samples from population A and 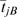 to be the equivalent for population B, it can be shown that
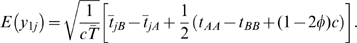
(23)An important implication of Equation 23 is that if the additional sample is the result of an admixture event between the two populations with a fraction 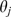 of its genome coming from population A then it follows that the location of the sample on the first PC is
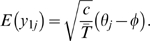
(24)In words, the admixture proportion of the individual can be directly inferred from their relative position along the first PC from the two source populations.

There are three important points to note when applying this result. First, only if the admixture event was very recent are the source populations likely to be available. Rather samples may be available for descendants of these source populations. Consequently, the average divergence between the population A part of an individuals genome and other samples from population A might typically be greater than for two samples taken directly from population A. However, this effect is likely to be very similar for the two source populations and, given Equation 23, these effects largely cancel out.

The second point to note is that if samples are admixed between more than two populations, the result generalises so that an individual whose genome is derived from several source populations will have a projected position (along each significant PC) defined by the weighted sum of the positions of its source populations. Informally, the result arises because of the linearity in Equation 22. Those parts of the genome with ancestry from a given population will have a PC projection that matches samples taken directly from the source population. If there is mixed ancestry, the effect is simply to average the PC projections.

Finally, it is important to note that projection of non-admixed individuals can also lead to their location being intermediate between the two original populations. For example, samples from a third population that either diverged from population A since the split with population B or that come from a population that diverged before the A/B split will (in both cases) be projected between the locations of samples from populations A and B. It may, however, be possible to distinguish between such cases by carrying out PCA on all data combined.

### PCA carried out on admixed individuals can identify relative admixture proportion in the absence of source populations

As has already been shown through simulation [1], PCA carried out on samples that are the result of admixture events can identify admixed samples as lying along the axes between the two or more source populations, even if one or more of the source populations are absent. The results above shed some light onto when such analyses are expected to work and when they will fail.

Consider a sample of individuals who are the result of an historical admixture event between two populations A and B. In order to define the matrix 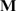 for this sample it is necessary to know which part of their genome is derived from each of the source populations. Let 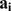 be a series of indicator functions for each of the 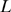 SNPs in individual 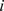 that takes value 1 if that part of the individual's genome was derived from population A and 0 if it was derived from population B. The value 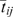 can be obtained by comparing the value of 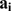 and 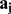 at each position and adding up the relevant contribution from each of 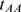, 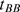 and 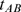. Note that here the achieved ancestry proportions are being used rather than their expectation under some model (which might be the same for all samples).

**Figure 4:**
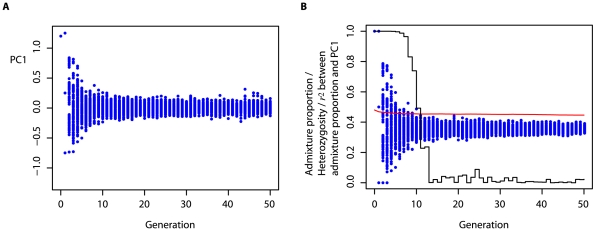
Identification of admixture proportions without source populations. Initially an admixed population is formed by random mating from two populations, each fixed for a different allele at each locus with 40% contribution from one population. In the simulated population there are 1000 individuals, each of which has 20 chromosomes with 50 markers each, a genetic map length of 1 per chromosome and a uniform recombination rate. Subsequent generations are formed by random mating of the ancestral population. (A) Projections of 100 randomly chosen samples on the first PC over time show a decay in the fraction of variance explained by the first PC (note that the total variance in the population decays little over the time-scale of the simulation). (B) Admxiture proportions for the same individuals as in part A (blue points) as well as the everage heterozygosity (red line) and the fraction of the variance in PC1 explained by admixture proportions (black line). While there is a strong association between admixture proportion and location on PC1 for the first few generations, after 15 generations recombination has eliminated any signal, even though there is still strong admixture LD between nearby markers (data not shown).

Given these considerations there are two situations under which none of the structure between the two source populations is expected to be reflected in the matrix 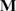. First, all individuals could have the same vector 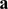, which could occur if the admixture event were ancient and involved relatively few individuals such that the source population at every point in the genome were fixed (note this does not mean that there is no variation, simply that all individuals at this location have an ancestry from the same population). Second, individuals have different ancestry vectors, but the average value is the same for all individuals and the admixture chunks have been sufficiently broken up through historical recombination such that everyone is equally related to everyone else. Again, this scenario could occur if the admixture result were ancient. Note that all individuals having the same average ancestry proportions is, by itself, not sufficient to create this problem. To examine the rate at which admixture signal is lost, an admixed population was simulated forward in time and the projections of samples on the first PC were followed, along with the correlation between PC projection and individual ancestry. As shown in Figure 4, in which the population is chosen to have parameters comparable to humans, the initially strong correlation between ancestry proportion and location on the first PC is rapidly lost such that after only 15 generations there is essentially no signal remaining, even though locally within the genome admixture chunks are still very clear (i.e. there is still admixture LD) after 50 generations.

## Discussion

The primary result of this paper is that the locations of samples on the principal components identified from genome-wide data on genetic variation can be predicted from an understanding of the average coalescent time for pairs of samples. This gives a direct route to understanding the influence various demographic scenarios can have on the relationships between samples identified from PCA and how PCA can be used to make inference about processes of interest such as admixture. However, the results also demonstrate the way in which sampling schemes can influence PC projections and how similar projections can arise from very different demographic scenarios. Consequently, using these results to motivate inference from PCA about underlying demographic process may prove difficult.

**Figure 5:**
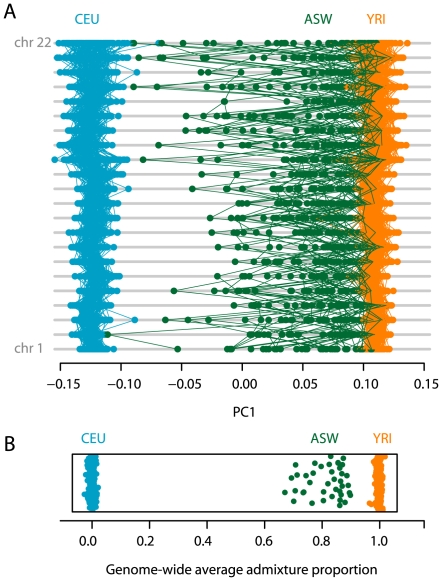
Admixture proportions inferred from PCA projections. (A) For each of the autosomes (chromosome 1 is the lowest) the points indicate the locations of sampled haplotypes (the transmitted and untransmitted haplotypes inferred from trios) on the first principal component (each chromosome is analysed separately; blue = CEU, orange = YRI, green = ASW). Importantly, PCA is carried out only on the haplotypes from CEU and YRI and all samples are subsequently projected onto the first PC identified from this analysis. Lines connect the transmitted (or untransmitted) haplotypes for each individual across chromosomes. Note the uniformity of the locations of samples on the first PC for CEU and YRI. Individual chromosomes within the ASW, however, show a great range of locations on the first PC. (B) The genome-wide admixture proportions (separately for transmitted and untransmitted chromosomes) can be inferred directly from the location of admixed samples on the first PC between the two source populations. Colours are as for (A). The vertical spacing of points is arbitrary.

There are, however, situations in which PCA can be used to infer demographic parameters directly. For example, in cases of simple two- or three-way admixture, where populations close to the source populations can be identified and sampled from, estimation of admixture proportions can be achieved from projecting samples onto the PCs identified from the source populations. To illustrate this, Figure 5 shows the inferred ancestry proportions for a set of haplotypes (estimated from trio data) in 20 African Americans collected as part of the HapMap3 project. In this analysis, haplotypes (also inferred from trios) from the European ancestry population in Utah (CEU) and the Yoruba in Nigeria (YRI) are used to represent the source populations (note, as discussed above, the requirement is not that these *are* the source populations, simply that they are closely related to the source populations). By analysing each chromosome separately it can be shown that while each individual's average ancestry proportion across the genome is fairly constant (typically 70–90% African), there is considerable variation at the level of individual chromosomes, with some chromosomes appearing essentially European (for some individuals) and others essentially African (no chromosome shows an overall tendency to come from one population). Such information could be informative about processes such as the level of assortative mating and the rate of ongoing admixture.

**Figure 6:**
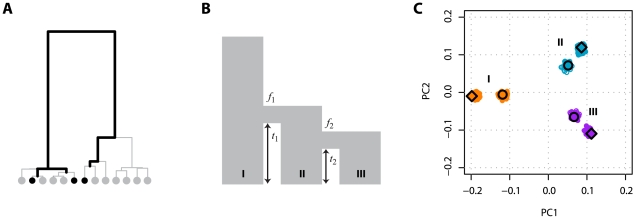
The effect of SNP ascertainment on PCA projection. (A) In the joint genealogy of the ascertainment (black circles) and genotyped samples (grey circles), only mutations occurring on the intersection of the two genealogies (shown in black) will be detected in both samples. For small discovery panels and large experimental samples, this may be considerably less than half the total genealogy length. (B) Model used to simulate data from three populations linked by two vicariance events, each of which is associated with a bottleneck; the model is an approximation to the demographic history of the HapMap populations [17],[18]. In the simulations 100 haploid genomes with 10,000 unlinked loci were sampled from each population and the parameters are 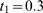, 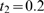, 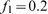, 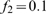, where 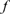 is the bottleneck strength measured as the probability that two lineages entering the bottleneck have coalesced by its end (the bottleneck is instantaneous in real time). All populations have the same effective population size. (C) PCA of the simulated data (small open circles) shows strong agreement with results obtained from analytical consideration of the expected coalescence times (large circles). When only those SNPs that have been discovered in a small panel are considered (here modelled as 4, 8, and 4 additional samples from populations I, II, and III respectively) the principal effect is to scale the locations of the samples on the first two PCs (small filled circles) by a factor of approximately 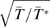 (large diamonds).

One important issue in the application of these ideas to the analysis of empirical data is the extent to which SNP ascertainment will influence outcome. SNP discovery in a small panel will typically lead to the under-representation of rare SNPs in the genotyped data and, depending on the geographical distribution of the samples used for discovery, can also lead to biases in the representation of variation from different areas. The quantities in Equation 8 are therefore conditional not just on segregation in the genotyped sample, but also on segregation within the SNP discovery panel. Consider the joint genealogy of the genotyped and discovery samples shown in Figure 6A. The probability that a pair of samples, 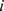 and 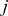 share a derived mutation (in the genotyped samples) that also lies on the subtree of the discovery samples, 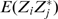 is
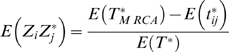
(25)where 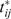 is the first time at which the common ancestor of the samples 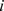 and 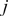 is also a common ancestor of at least one of the discovery panel samples (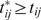), 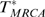 is the time to the more recent of the discovery or sample MRCAs and 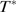 is the total time of the intersection between the discovery and genotyped samples' genealogies (Figure 6A). It follows that the equivalent expression for Equation 10 with SNP ascertainment will typically be larger than without SNP ascertainment because 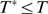 whereas the differences in the numerators will largely cancel each other out. Consequently, it is expected that, except for very strongly biased SNP discovery (e.g. a sample of two from one of a series of very divergent populations), that PCA projections from genotype data will be similar to PCA projections from resequencing data, but will typically be larger in magnitude (if the matrix 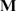 is normalized by the number of SNPs) by a factor 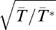; a result confirmed by simulation (Figure 6B and 6C). For the example shown, this result holds even under the most extreme ascertainment scheme of two discovery samples from a single population. In short, SNP ascertainment will tend to have a simple and predictable effect on PC projections that has little influence on the relative placing of samples.

Finally, it is worth pointing out that because PCA effectively summarizes structure in the matrix of average pairwise coalescent times, but in a manner that is influenced by sample composition, more direct inferences can potentially be made from the matrix of pairwise differences (which are trivially related to pairwise coalescent times). This is not to say that eigenvalue analysis of the pairwise distance matrix will correct for the effects of biased sampling demonstrated in Figure 3. However, while readily-available alternatives to PCA, such as multidimensional scaling, seem to have properties similar to PCA, it is possible to envisage non-parametric methods for analysing the matrix of pairwise differences that identify structure without being influenced by sample size.

## Methods

Coalescent simulations were carried out using scripts written by the author in the R language (www.r-project.org) and available on request. Principal component analysis of simulated data was carried out using the R function eigen. Phased haplotypes from the International HapMap Project (HapMap3 release 2) were used in the analysis of the CEU, YRI and ASW population (see ftp://ftp.hapmap.org/hapmap/phasing/2009-02_phaseIII/HapMap3_r2/).
